# Activity of Ozonated Water in Sterilising and Disinfecting Dental Unit Water Pipelines System: A Comparative Study

**DOI:** 10.3290/j.ohpd.b2580291

**Published:** 2022-01-20

**Authors:** Kiran Kumar Ganji, Sultan Meteb Alshammari, Munahi Abdullah Rushdallah, Amany A. Ghazy, Ibrahim Taher, Ahmed E. Taha, Rakhi Issrani, Muhannad Ahmed Nazal Alhazmi

**Affiliations:** a Assistant Professor, Department of Preventive Dentistry, College of Dentistry, Jouf University, Al Jouf, Saudi Arabia. Study concept, supervision, literature review, wrote and critically reviewed the manuscript.; b Dentist, Department of Preventive Dentistry, College of Dentistry, Jouf University, Al Jouf, Saudi Arabia. Study concept, data collection/processing, wrote and critically reviewed the manuscript.; c Dentist, Department of Preventive Dentistry, College of Dentistry, Jouf University, Al Jouf, Saudi Arabia. Study design, supervision, critically reviewed the manuscript.; d Associate Professor, Medical Microbiology and Immunology unit, Department of Pathology, College of Medicine, Jouf University, Saudi Arabia. Study design, microbiological testing, analysis/interpretation of results, literature review, wrote and critically reviewed the manuscript.; e Professor, Medical Microbiology and Immunology unit, Department of Pathology, College of Medicine, Jouf University, Saudi Arabia. Study design and supervision, microbiological testing, analysis/interpretation of results, literature review, wrote and critically reviewed the manuscript.; f Assistant Professor, Medical Microbiology and Immunology unit, Department of Pathology, College of Medicine, Jouf University, Saudi Arabia. Study design and supervision, microbiological testing, analysis/interpretation of results, literature review, wrote and critically reviewed the manuscript.; g Lecturer, Department of Preventive Dentistry, College of Dentistry, Jouf University, Al Jouf, Saudi Arabia. Study concept, critically reviewed the manuscript.; h Dentist, Department of Preventive Dentistry, College of Dentistry, Jouf University, Al Jouf, Saudi Arabia. Study design, data collection/processing, wrote and critically reviewed the manuscript.

**Keywords:** biofilms, dental-unit water pipelines, disinfection, microbial contamination, ozonated water

## Abstract

**Purpose:**

A number of disinfectants and sanitisers are used in dentistry, and there are numerous commercial solutions available. Nonetheless, because each cleaning solution has its own set of indications and limits, there is no one-size-fits-all approach for processing all types of dental equipment. Functional water, such as electrolysed hypochlorite microbubbled water, efficiently eliminates and sterilises biofilms. The objective of the study was to evaluate whether ozonated water could be used to sterilise and disinfect dental-unit water pipelines (DUWP) that had been contaminated with micro-organisms, including Gram-positive and Gram-negative bacilli and cocci.

**Materials and Methods:**

Three different groups were formed: group A – ozonated water (Cantoosh); group B – 1% povidine iodine; and group C: conventional distilled water. Group A was the test group, group B the control group, and group C was the positive control group. The water sterilising system was replaced with the appropriate sterilising agent as per the allocated group classification, with 2 min of purging, so that the complete DUWP was filled with the water sterilising system. Samples were collected and analysed, along with a 2-min purge after 24 h, 7 days and 21 days, at the 3 outlet (OL) points: the 3-way syringe at the dental tray(OL1), the cup filler (OL2), and the 3-way syringe of the assistant zone (OL3). Repeated measures ANOVA was used to test for statistical significance between colony-forming units of control and experimental groups (p < 0.05).

**Results:**

The cup filler yielded higher counts than did the 3-way syringe at the dental tray (OL1) (6.40 and 8.05 on the log scale, respectively). A statistically significant difference in the CFUs was also observed between samples taken after 24 h vs 21 days between groups A, B and C.

**Conclusion:**

The findings showed that exposing DUWP tube systems to ozonated water for an extended length of time drastically lowered the number of microorganisms adhering to their surfaces.

Dental unit water pipelines (DUWP) are systems that supply all the instruments (e.g. handpieces, ultrasonic scaler, and three-way syringes) with the water needed during dental procedures. The DUWPs are connected to water bottles at the dental chair or to a municipal water source. This system must be of good quality, free from harmful water-borne micro-organisms. Otherwise, it can be a potential infection source for those regularly exposed to the water or its aerosols, that is, dentists, their assistants, and the treated patients, especially immunocompromised individuals.^20^ As shown in a previous study,^5^ health-associated microbial communities had more varied functional activities than disease-associated microbial communities, despite substantial interpatient heterogeneity in microbiome composition. Extracellular matrix is vital for microbial biofilms. The extracellular matrix maintains the spatial arrangement of cells and coordinates cellular activities across microbial biofilms, such as dental plaque.^17^ They are often structured into macromolecular complexes and connected with microbial cell surfaces inside the biofilm. Thus, controlling the matrix is essential for oral health.^17^ Blake^4^ reported the presence of bacteria inside biofilms in DUWPs, water, and aerosols created by dental procedures for the first time in 1963. Thus, the water must be of high quality, as defined by the limits recommended for human consumption.^40^ The standard levels set by the American Dental Association for dental-unit water for nonsurgical procedure is < 5 x 10^2^ colony-forming units (CFUs), and it is recommended to use sterile saline or sterile water as a coolant or irrigant for surgical procedures.^25^ However, contamination levels as high as 10^5^ CFU/ml have been found.^18,25^ An 81-year-old female patient died in Italy after contracting Legionnaire’s disease upon exposure to contaminated water from the internal dental-unit waterline.^30^ Serological investigations have shown that the prevalence of antibodies to L. pneumophila is higher among employees of dental offices and dentists, suggesting that this group of professionals may be at risk of infection.^38^ Waterlines have been contaminated by bacteria such as *Pseudomonas aeruginosa, Aeromonas hydrophila, Staphylococcus aureus, Klebsiella pneumoniae, Bacillus spp., Escherichia coli, Enterobacter spp.,* and *Streptococcus spp.*^26^ If bacterial concentrations in dental-unit reservoirs exceed safe levels, disinfection procedures are necessary.^37^ Substantial rates of contamination (1.8 x 10^6^ CFU/ml) have been found in DUWPs, which emphasises the necessity to develop national standards. It is necessary to sanitise DUWPs regularly and utilise a cleaner water supply.^19^ A recent report showed that dental surgeons’ knowledge must be increased to reduce the risk of infectious disease caused by drinking water in DUWP systems.^31^

In dentistry, a variety of disinfectants and sanitisers are employed. Numerous commercial solutions are available, but each cleaning solution has its own set of indications and limitations. Furthermore, there is no one-size-fits-all approach for processing all types of dental equipment. In the current context, hydrogen peroxide at 3% is a good candidate for periodic household microfilled water-dispenser disinfection, given its low cost and easy accessibility.^44^ Alternative means of sterilising and disinfecting dental equipment are constantly being researched, and the use of ozone to disinfect dental equipment is of particular importance.^6,9,39^

Ozone is thought to have notable antibacterial properties because it may oxidise amino acids and damage proteins found in the cell membranes of microorganisms. Ozone has been applied in wound therapy and microbe control in drinking water, the food sector, and medicine.^8,10^ Several authors have highlighted the use of ozone in dentistry, whether as an oil or ozonated water.^24^ Ozaki et al^27^ reported that using functional water, such as electrolysed hypochlorite micro-bubbled water, efficiently eliminated and sterilised biofilms. However, the literature on the use of ozonated water for sanitising DUWPs is scant. The goal of this investigation was to evaluate whether ozonated water could be used to sterilise and disinfect DUWP systems that had been contaminated with gram-positive and gram-negative water-borne bacteria.

## Materials and Methods

An in vitro double-blinded study was conducted at outpatient departmental clinics of the College of Dentistry, Jouf University, Kingdom of Saudi Arabia. The study was approved by the institutional local committee for bioethics (Jouf University, Kingdom of Saudi Arabia). For the purposes of this study, contaminated water of DUWPs was obtained from 3 dental chair units (Adec; Newberg, OR, USA) which were not routinely used for dental procedures. After confirming similar levels of contamination, the three dental units were allocated to three different groups. Each group was subjected to one of the three water-sterilising protocols: group A (test group): ozonated water (Cantoosh; Riyadh, Kingdom of Saudi Arabia), group B (control): 1% povidone iodine: group C (positive control): conventional distilled water. All 3 dental units were subjected to the same daily maintenance, which followed professional guidelines and was documented on a separate traceability sheet.

### Preparation of Disinfecting Solutions (groups A and B)

The prototype equipment, which was employed in our earlier investigation,^3^ was employed to create the oxygen-ozone combination and ozonated water. Ozonated water (sodium hypochlorite) for group A was obtained with the help of Cantoosh, based on the electrogenerated hypochlorite concept. It is composed of a platinum base to which crystallized salt and water are added.^21^ As per the manufacturer’s instructions, it was filled with water, leaving a 2.5-cm space on top, followed by adding one spoonful salt, and was then connected for 5 min to an electrical unit producing ozonated water. 1% povidone-iodine solution for group B was prepared.

### Processing and Analysis of Collected Samples

**Fig 1:**
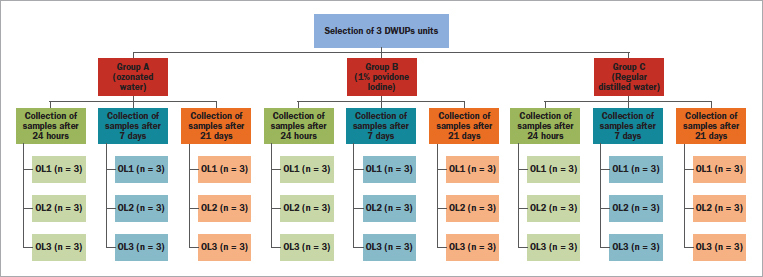
Schematic flowchart for process of randomization and collection of samples.

After 24 h of seeding time with the distilled water in the three selected DUWPs units, the output water samples were collected from these three DUWPs by a trained dental assistant blinded to sample collection (Fig 1). The microbiologists who analysed the samples were blinded as to sample group. The three outlets are referred to as sampling points of the DUWP system, consisting of the 3-way syringe at instrument tray unit outlet (OL1), cup filler (OL2), and 3-way syringe of the assistant zone (OL3). Water samples were obtained at these points after conducting the respective water-sterilising protocols. In the same manner, water sampling was repeated after an 24 h, 7 days and 21 days to evaluate the sterilising efficacy and rate of reinfection.

### Microbiological Analysis and Culture Conditions

To counteract the possible bacterial contamination risk in the DUWP system, it was essential to evaluate the total bacterial load and identify the most probable contaminants. In the current study, different water samples were analysed using standard microbiological procedures.

A total of 81 samples were collected. Each sample consisted of 50 ml of treated water; 10 ml aliquots were centrifuged (Universal 320R-Hettich Zentrifugen; Tuttlingen, Germany) at 1000 rpm for 10 min, and the pellet was collected and emulsified in 1 ml of sterile normal saline. Gram-^11,23^ and directly ZN^34^-stained films were prepared and examined microscopically to detect any gram-positive and gram-negative bacteria as well as mycobacteria.

The total viable bacterial count was performed by spreading 100 ul of each sample on duplicate blood agar (BA) (Oxoid; Basingstoke, UK) and MacConkey agar (MAC plates), and Sabouraud dextrose agar plates (SDA, Oxoid) to isolate fungi. The BA and MAC plates were incubated for 24 to 48 h at 37°C (Memmert; Büchenbach, Germany), while the SDA plates were incubated for 7 days at 30°C. After a sufficient length of incubation, microbial growth was counted as colony-forming units per ml (CFU/ml).^16^ All bacterial isolates were identified using the Vitek-2 compact system (BioMérieux; Marcy l’Étoile, France).

Reporting of acid-fast bacilli (AFB) observed in stained smears was based on the K. Eisenach classification (Stinson et al^36^), where negative means no bacteria, 1+ indicates the presence of 1-6 AFB in one field, 2+ indicates the presence of 7-60 AFB, +3 indicates > 60 AFB in one field.

## Results

**Fig 2:**
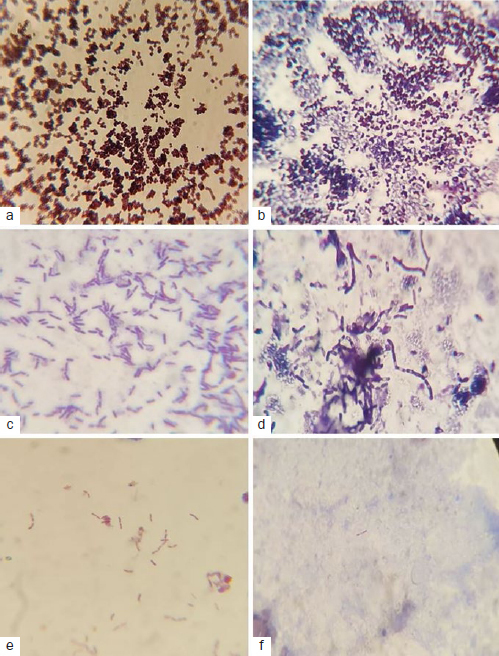
Microscope images of Gram-positive diplococci (a, b), Gram-positive spore-forming bacilli (c, d) and ZN-mycobacteria (e, f).

A total of 81 samples were collected from the respective sampling points of DUWPs. The sterilising efficacy of all units is presented in Tables 1 to 3. Water contamination levels as high as 2 x 10^4^ CFU/ml were found in samples taken 24 h after sterilising treatment of each unit (Fig 2). At all sample collection times (24 h, 7 days, and 21 days), ozonated water (group A) showed a greater antimicrobial effect against Gram-positive and Gram-negative strains compared to the control group and positive control group.

**Table 1:** Microbial load (CFU/ml) of group A, B and C samples after 24 h of sterilisation

bacterial examination	Group A, Ozonated water (n = 9)			Group B, control (n = 9)			Group C, positive control (n = 9)		
	OL1 (n = 3)	OL2 (n = 3)	OL3 (n = 3)	OL1 (n = 3)	OL2 (n = 3)	OL3 (n = 3)	OL1 (n = 3)	OL2 (n = 3)	OL3 (n = 3)
Direct ZN-stained film	0	+	0	+	+	+	+++	+	++
Direct Gram-stained film	0	0	0	G+ cocci	0	G+ bacilli	G+ bacilli	0	G+ bacilli G+ cocci
Average total viable count (CFU/ml)	0	2 x 10	0	1 x 10^3^	1 x 10^3^	1 x 10^3^	3.1 x 10^3^	4 x 10^3^	2.1 x 10^3^
bacterial culture results	0	G+ spore-forming bacilli	0	G+ spore-forming bacilli, G+ Diplococci	G+ spore-forming bacilli	G+ spore-forming bacilli	G+ spore-forming bacilli, G+ diplococci	G+ spore-forming bacilli, G+ diplococci	G+ spore-forming bacilli, G+ diplococci
Fungal culture results	0	0	0	0	0	0	0	0	0

G+: Gram positive; group A: Ozonated water (Cantoosh); group B: water treated by 1% povidone Iodine; group C: conventional distilled water. OL1: sample collected from 3-way syringe at the instrument tray unit outlet; OL2: sample collected from cup filler outlet; OL3: sample collected from 3-way syringe of assistant zone outlet. For directly ZN-stained film: + (1-6 AFB), ++ (7-60 AFB), and +++ (> 60 AFB).

**Table 2:** Microbial load (CFU/ml) of group A, B and C samples after 7 days of sterilisation

Bacterial examination	Group A, Ozonated water (n = 9)			Group B, control(n = 9)			Group C, +ve control(n = 9)		
	OL1 (n = 3)	OL2 (n = 3)	OL3 (n = 3)	OL1 (n = 3)	OL2 (n = 3)	OL3 (n = 3)	OL1 (n = 3)	OL2 (n = 3)	OL3 (n = 3)
Direct ZN-stained film	+	0	+	+	0	0	+	+	+
Direct Gram-stained film	0	0	0	G- bacilli	0	G+ Bacilli G+ cocci	G+ cocci	G+ Bacilli	G+ cocci
Average total viable count (Cfu/ml)	0	0	0	2 x 10^4^	0	6.4 x 10^3^	7 x 10^3^	8 x 10^3^	7.1 x 10^3^
Bacterial culture results	0	0	0	G- bacilli	0	G+ spore-forming bacilli, G+ diplococci	G+ spore-forming bacilli, G+ diplococci	G+ spore-forming bacilli, G+ diplococci	G+ spore-forming bacilli, G+ diplococci
Fungal Culture results	0	0	0	0	0	0	0	0	0

G+: Gram positive; G-: Gram negative; group A: ozonated water (Cantoosh); group B: water treated by 1% povidone Iodine; group C: conventional distilled water. OL1: sample collected from 3-way syringe at instrument tray unit outlet; OL2: sample collected from cup filler outlet; OL3: sample collected from 3-way syringe of assistant zone outlet. For directly ZN-stained film: + (1-6 AFB), ++ (7-60 AFB), and +++ (> 60 AFB).

**Table 3:** Microbial load (CFU/ml) of group A, B and C samples after 21 days of sterilisation

Bacterial examination	Group A, Ozonated water (n = 9)			Group B, control (n = 9)			Group C, +ve control (n = 9)		
	OL1 (n = 3)	OL2 (n = 3)	OL3 (n = 3)	OL1 (n = 3)	OL2 (n = 3)	OL3 (n = 3)	OL1 (n = 3)	OL2 (n = 3)	OL3 (n = 3)
Direct ZN-stained film	0	0	0	0	0	0	0	0	0
Direct Gram-stained film	G+ Bacilli	0	0	0	G+ cocci	0	G+ cocci	G+ Bacilli	G+ cocci
Average total viable count (Cfu/ml)	4.4 x 10^2^	0	0	1.7 x 10^3^	4 x 10^3^	5 x 10^3^	2.2 x 10^3^	8.4 x 10^3^	2 x 10^4^
Bacterial culture results	G+ spore-forming bacilli	0	0	G+ spore-forming bacilli	G+ diplococci	G+ spore-forming bacilli	G+ diplococci	G+ spore-forming bacilli	G+ diplococci
Fungal Culture results	0	0	0	0	0	0	0	0	0

G+: Gram positive, G-: Gram negative; group A: ozonated water (Cantoosh); group B: water treated by 1% povidone Iodine; group C: conventional distilled water. OL1: sample collected from 3-way syringe at instrument tray unit outlet; OL2: sample collected from cup filler outlet; OL3: sample collected from 3-way syringe of assistant zone outlet. For directly ZN-stained film: + (1-6 AFB), ++ (7-60 AFB), and +++ (> 60 AFB).

**Fig 3:**
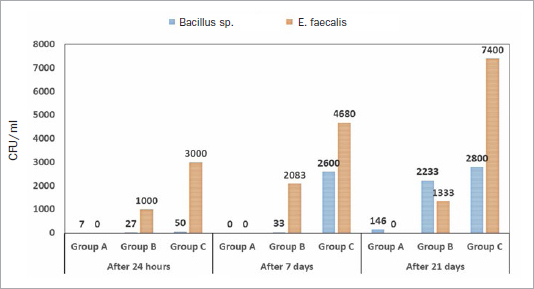
Average counts of *Bacillus* sp*.* and *E. faecalis* per group.

The number of bacterial colonies varied dramatically depending on the source of the water, and the growth conditions examined, ranging from 0 to over 2 x 10^4^ CFU/ml. Colony counts differed statistically significantly on BA, MAC, and SDA media after the incubation period (Fig 3). The counts on BA and MAC media were greater than those on SDA media. The bacteria in the positive control group were viable; however, there were no colony-forming units in the negative control group (Table 1–3). Bacterial identification with Vitek-2 (Biomerieux) revealed *Bacillus cereus, Enterococcus faecalis, Staphylococcus lentus, Kocuria rosea,* gram-negative non-fermenting bacilli, and some unidentified species (Table 4). However, no CFUs of *Pseudomonas aeruginosa, Legionella pneumophila* or fungi were found in the water samples. The cup filler yielded more CFUs than the 3-way syringe at the dental tray (6.40 and 8.05 on the log scale, respectively), which was a statistically significant difference (p < 0.05). A statistically significant difference in the CFUs was also observed between samples taken after 24 h and 21 days. Repeated-measures ANOVA revealed a statistically significant difference in CFUs between the control and experimental groups (p < 0.05). Regarding the AFB, directly ZN-stained films revealed variable degrees of mycobacterial contamination of water samples (Table 1–3).

**Table 4:** Isolates identified by the Vitek-2 compact system

Isolate identified	Number of isolates								
	After 24 h			After 7 days			After 21 days		
	Group A	Group B	Group C	Group A	Group B	Group C	Group A	Group B	Group C
*Enterococcus faecalis*	0	1	3	0	1	2	0	1	2
*Bacillus cereus*	1	3	3	0	1	3	1	2	1
*Non-fermenting Gram negative bacilli*	0	0	0	0	1	0	0	0	0
*Kocuria rosea*	0	0	1	0	0	0	0	0	1
*Staphylococcus lentus*	0	0	0	0	0	1	0	0	1

Group A: ozonated water (Cantoosh); group B: water treated with 1% povidone Iodine; group C: conventional distilled water.

## Discussion

Gaseous ozone and aqueous ozone solutions have a well-documented bactericidal effect on planktonic bacterial cells. At the same time, little research has been done on the effects of ozone on bacterial biofilms and bacterial growth. Biofilms help bacteria colonise and develop on biotic (tissue) surfaces, including the oral mucosa, ureters, and lungs, as well as abiotic surfaces such as catheters and implants. Freshly ozonated water was shown to be bactericidal against biofilms created by clinical strains of *S. aureus*, as well as to produce a significant decrease in the number of viable cells in *P. aeruginosa* biofilms. However, biofilms of *P. aeruginosa* were more tolerant to ozonated water.

In this investigation, ozonated water was shown to be effective against many strains of both Gram-positive and Gram-negative bacteria. The antibacterial activity remained for a period of 21 days following treatment. These findings were in agreement with Ozaki et al,^27^ who proved that biofilms might be removed and sterilised using ozonated water. One of the most significant benefits of using ozonated water as a disinfectant is its sporicidal action. Similarly, Makky et al^22^ observed that exposure to ozonated water for 10 min at 10 mg/l inactivated *B. atrophaeus* spores 99.9% of the time. One of the reasons for such encouraging results from ozonated water could be explained by the different systems’ modes of action. The differences could be in part due to the water flow detaching bacteria from the biofilm, which would allow the chemical disinfectant to kill these planktonic bacteria and outer layers of biofilm. However, chemical disinfection, has little effect on the deep layers of biofilms. On the other hand, biofilm-producing bacteria are significantly more resistant to disinfectants than planktonic bacteria, and biofilm bacteria may continue to function as a reservoir for bacterial contamination inside the DUWPs. Jean Barbeau^1^ thought that bacteria in the water samples obtained from DUWPs basically stem from biofilm formed inside the tubes of DUWPs. He added that the majority of the bacterial species discovered belong to the families of soil and aquatic bacteria, where pigmented bacteria were also seen.^1^

The bacterial concentrations reported in DUWPs varied from 0 to 1.52 x 10^6^ CFU/ml, according to Souza-Gugelmin et al,^35^ who incubated the samples for 48 h at 37°C and determined the bacterial growth. Based on the safety threshold suggested by Souza-Gugelmin et al,^35^ most of the reservoirs’ water was polluted with bacteria, whereas bacterial contamination in other, similar studies was far above the permissible amount.^12^ Although all of the reservoirs in our analysis were contaminated, the average contamination level was substantially higher than the highest level found by Depaola et al.^12^ However, in the study by Tuttlebee et al,^40^ the average bacterial count was 6.6 x 10^4^, which was lower than that of our study.

Mycobacteria are opportunistic microbes found in soil and water. In the present study, variable degrees of mycobacterial contamination of water samples were detected by directly ZN-stained films. Many investigations have indicated the presence of mycobacteria in water samples obtained from DUWPs, which poses a risk of illness.^32,29^ Recent reports from the United States^15,33^ and Venezuela^28^ indicate that these deadly bacteria have caused outbreaks following dental treatment. In 2015, 24 children in Georgia (USA) were diagnosed with severe *Mycobacterium abscessus* infection after a pulpotomy procedure; 17 of them required antibiotic therapy and surgical excision of the infected tissue. *Mycobacterium abscessus* was isolated from samples of all water sources used during the procedure.^15^ In California, the largest odontogenic mycobacterial outbreak described to date was reported, afflicting at least 71 children with abnormalities of the maxilla or mandible. The same species of nontuberculous mycobacteria (NTM) were isolated from all DUWPs used for the treatment of the children.^33^ Moreover, in Venezuela, severe *M. peregrinum, M. fortuitum,* and *M. abscessus* infections were diagnosed in 3 adult patients after dental procedures due to contamination of DUWPs with these mycobacterial species.^28^

In the United Kingdom, 95% of DUWP water tests exceeded the European Union drinking water limits for microbial load, with mycobacterium species contaminating 10% of the water samples.^41^ The pathogenic potential of the isolates is unknown because these isolates were not identified.^41^ In Ecuador, the water quality of DUWPs in the cities of Caracas and Quito was assessed. Results showed that 56% and 3% of the DUWPs in Caracas and Quito, respectively, yielded NTM species, ranging up to 1000 CFU/ml. Additionally, the authors noted that mycobacteria are more resistant to disinfection techniques than other bacteria.^7^ All studies testing mycobacterial contamination of the DUWPs show that a high mycobacterial load may be inhaled, swallowed, or inoculated into the oral wound during any dental procedure, with the possibility of mycobacterial sensitisation, colonisation, or infection.^29^ Concerning adequate antimycobacterial disinfectants for DUWPs, no recommendations are available in the existing guidelines.^7^

Dentists must use sterile water or water of suitable microbiological purity to keep patients safe, especially while performing surgical operations and/or treating special groups of patients, such as the elderly or immunocompromised. Contamination of DUWPs with *Bacillus cereus, Enterococcus faecalis*, *Staphylococcus lentus*, *Kocuria rosea*, gram-negative non-fermenting bacilli, some unidentified species, and mycobacteria necessitates regular disinfection of DUWPs and monitoring the bacterial content of the water. The presence of mycobacteria in the water indicates that the water should be tested after disinfection of the DUWPs, because low-level disinfectants eliminate most contaminating bacteria but cannot remove mycobacteria, resulting in their accumulation in water.

Results from our study serve as a warning for dental offices about the necessity of implementing biosecurity and infection control measures. Dentists should recognise the limitations of existing disinfecting systems for DUWPs. According to the findings of this investigation, samples obtained from the cup filler had the highest number of CFUs of micro-organisms, followed by a 3-way air-water syringe. These findings contradicted those of James et al,^18^ who found that samples taken from the handpiece had the most CFUs, followed by water-air syringe and cup filler. These findings indicate that the handpiece and other DUWP components produce a biofilm, and that water flowing down biofilm-coated waterlines will help in the creation of microbicides. Because the water in the current study’s DUWPs was stagnant, frequent periods of water stagnation in DUWPs (related to idle phases during the day, evenings, nights, weekends, and holidays), as well as the properties of the plastics used in DUWPs construction, may encourage the attachment and colonisation of biofilm-forming micro-organisms. The internal diameter of most plastic dental tubing ranges from 8 mm to 16 mm. Narrow-bore tubing has a very large surface-area-to-volume ratio compared to tubing of larger bores.^43^

One of the major limitations of the ozonated water is that, once made, the storage of ozone is not possible, since ozonated water has a limited half-life, and residual ozone is active in water for a maximum of 8 h.^2^ As a result, when ozone is needed for disinfection, it must be created on-site.^42^ As a result, flowing water in the DUWPs creates an infection risk. To establish the system’s potential effectiveness, long-term research is necessary. Nonetheless, in the face of other proven and commercially available waterline disinfection systems for dental units and for the safety of patients, it would be unethical to continue employing systems of doubtful efficacy. We did not test the antimicrobial susceptibility and resistance of the isolates, as this would have been beyond the scope of the study. Furthermore, we did not look for protozoa, enteric viruses, or bacterial spores, which can also contaminate the waterlines and are more resistant to disinfectants.

## Conclusion

The findings of the current study showed that exposing DUWPs to ozonated water for an extended length of time was successful in drastically lowering the number of micro-organisms adhering to their surfaces. In most DUWPs, we found bacterial pathogens, including mycobacteria, which may pose a threat to human health. The discovery of mycobacteria in DUWPs demonstrates the necessity of performing water quality testing for these lethal species.

## References

[ref1] Barbeau J, Tanguay R, Faucher E, Avezard C, Trudel L, Côté L (1996). Multiparametric analysis of waterline contamination in dental units. Appl Environ Microbiol.

[ref2] Bezirtzoglou E, Cretoiu S-M, Moldoveanu M, Alexopoulos A, Lazar V, Nakou M (2008). A quantitative approach to the effectiveness of ozone against microbiota organisms colonizing toothbrushes. J Dent.

[ref3] Bialoszewski D, Bocian E, Bukowska B, Czajkowska M, Sokol-Leszczynska B, Tyski S (2010). Antimicrobial activity of ozonated water. Med Sci Monit.

[ref4] Blake G (1963). The incidence and control of bacterial infection in dental spray reservoirs. Brit Dent J.

[ref5] Buduneli N (2021). Environmental factors and periodontal microbiome. Periodontol 2000.

[ref6] Cardoso MG, De Oliveira LD, Koga-Ito CY, Jorge AOC (2008). Effectiveness of ozonated water on Candida albicans, Enterococcus faecalis, and endotoxins in root canals. Oral surgery, oral medicine, oral pathology, oral radiology, and endodontology.

[ref7] Castellano Realpe OJ, Gutiérrez JC, Sierra DA, Pazmino Martinez LA, Prado Palacios YY, Echeverría G (2020). Dental unit waterlines in Quito and Caracas contaminated with nontuberculous mycobacteria: a potential health risk in dental practice. Int J Environ Res Public Health.

[ref8] Čehovin M, Medic A, Scheideler J, Mielcke J, Ried A, Kompare B (2017). Hydrodynamic cavitation in combination with the ozone, hydrogen peroxide and the UV-based advanced oxidation processes for the removal of natural organic matter from drinking water. Ultrason Sonochem.

[ref9] César J, Sumita TC, Junqueira JC, Jorge AOC, Do Rego MA (2012). Antimicrobial effects of ozonated water on the sanitization of dental instruments contaminated with E coli, S. aureus C. albicans or the spores of B. atrophaeus. J Infect Public Health.

[ref10] Chand R, Bremner DH, Namkung KC, Collier PJ, Gogate PR (2007). Water disinfection using the novel approach of ozone and a liquid whistle reactor. Biochem Engineer J.

[ref11] Coico R (2006). Gram staining. Curr Protoc Microbiol.

[ref12] Depaola LG, Mangan D, Mills SE, Costerton W, Barbeau J, Shearer B (2002). A review of the science regarding dental unit waterlines. JADA.

[ref13] Eggers M (2019). Infectious disease management and control with povidone iodine. Infectious diseases and therapy.

[ref14] Eggers M, Koburger-Janssen T, Eickmann M, Zorn J (2018). In vitro bactericidal and virucidal efficacy of povidone-iodine gargle/mouthwash against respiratory and oral tract pathogens. Infectious diseases and therapy.

[ref15] Hatzenbuehler LA, Tobin-D’angelo M, Drenzek C, Peralta G, Cranmer LC, Anderson EJ (2017). Pediatric dental clinic–associated outbreak of Mycobacterium abscessus infection. J Pediatr Infect Dis Soc.

[ref16] ISO Standard (1999). Water quality –Enumeration of culturable micro-organism –colony count by inoculation in a nutrient agar culture medium. International Organization for Standardization (EN ISO Standard 6222–1999).

[ref17] Jakubovics NS, Goodman SD, Mashburn-Warren L, Stafford GP, Cieplik F (2021). The dental plaque biofilm matrix. Periodontol 2000.

[ref18] James A, Shetty A, Hedge M, Bhandary S (2015). Detection and quantification of microorganisms in dental unit waterlines. J Dent Med Sci.

[ref19] Ji X-Y, Fei C-N, Zhang Y, Liu J, Liu H, Song J (2019). Three key factors influencing the bacterial contamination of dental unit waterlines: a 6-year survey from 2012 to 2017. Int Dent J.

[ref20] Lizzadro J, Mazzotta M, Girolamini L, Dormi A, Pellati T, Cristino S (2019). Comparison between two types of dental unit waterlines: How evaluation of microbiological contamination can support risk containment. Int J Environ Res Public Health.

[ref21] Locker J, Fitzgerald P, Sharp D (2014). Antibacterial validation of electrogenerated hypochlorite using carbon-based electrodes. Lett Appl Microbiol.

[ref22] Makky EA, Park G-S, Choi I-W, Cho S-I, Kim H (2011). Comparison of Fe (VI)(FeO42-) and ozone in inactivating Bacillus subtilis spores. Chemosphere.

[ref23] Moyes RB, Reynolds J, Breakwell DP (2009). Differential staining of bacteria: gram stain. Curr Protoc Microbiol.

[ref24] Nagayoshi M, Fukuizumi T, Kitamura C, Yano J, Terashita M, Nishihara T (2004). Efficacy of ozone on survival and permeability of oral microorganisms. Oral Microbiol Immunol.

[ref25] Offner D, Fioretti F, Musset A-M (2016). Contamination of dental unit waterlines: Assessment of three continuous water disinfection systems. BDJ open.

[ref26] Oleiwi R (2017). Bacterial contamination of dental unity water lines (DUWL) in Baghdad city. IOSR J Dent Med Sci.

[ref27] Ozaki M, Ohshima T, Mukumoto M, Konishi H, Hirashita A, Maeda N (2012). A study for biofilm removing and antimicrobial effects by microbubbled tap water and other functional water, electrolyzed hypochlorite water and ozonated water. Dent Mater J.

[ref28] Pérez-Alfonzo R, Brito LEP, Vergara MS, Damasco AR, Rodríguez PLM, Quintero CEK (2020). Odontogenic cutaneous sinus tracts due to infection with nontuberculous mycobacteria: a report of three cases. BMC Infect Dis.

[ref29] Porteous N, Redding S, Jorgensen J (2004). Isolation of non-tuberculosis mycobacteria in treated dental unit waterlines. Oral Surg Oral Med Oral Pathol Oral Radiol Endodontol.

[ref30] Ricci ML, Fontana S, Pinci F, Fiumana E, Pedna MF, Farolfi P (2012). Pneumonia associated with a dental unit waterline. Lancet.

[ref31] Robert A, Bousseau A, Costa D, Barbot V, Imbert C (2013). Are dentists enough aware of infectious risk associated with dental unit waterlines?. Bull Group Int Rech Sci Stomatol Odontol.

[ref32] Schulze-Röbbecke R, Feldmann C, Fischeder R, Janning B, Exner M, Wahl G (1995). Dental units: an environmental study of sources of potentially pathogenic mycobacteria. Tuber Lung Dis.

[ref33] Singh J, O’Donnell K, Ashouri N, Adler-Shohet FC, Nieves D, Tran MT (2018). 926. Outbreak of invasive nontuberculous mycobacterium (NTM) infections associated with a pediatric dental practice. Open Forum Infectious Diseases.

[ref34] Smith H, Mcdiarmid A, Smith A, Hinson A, Gilmour R (1989). An analysis of staining methods for the detection of Cryptosporidium spp. oocysts in water-related samples. Parasitol.

[ref35] Souza-Gugelmin MCMD, Lima CDT, Lima SNMD, Mian H, Ito IY (2003). Microbial contamination in dental unit waterlines. Braz Dent J.

[ref36] Stinson K, Eisenach K, Kayes S, Matsumoto M, Siddiqi S, Nakashima S (2014). Mycobacteriology laboratory manual. Global laboratory initiative, a working group of the stop TB partnership.

[ref37] Szymanska J (2007). Bacterial contamination of water in dental unit reservoirs. Ann Agric Environ Med.

[ref38] Szymanska J (2004). Risk of exposure to Legionella in dental practice. Ann Agric Environ Med.

[ref39] Thomas LP, Bebermeyer RD, Dickinson SK (2005). Methods of dental instrument processing, sterilization, and storage –a review. Tex Dent J.

[ref40] Tuttlebee C, O’Donnell M, Keane C, Russell R, Sullivan D, Falkiner F (2002). Effective control of dental chair unit waterline biofilm and marked reduction of bacterial contamination of output water using two peroxide-based disinfectants. J Hosp Infect.

[ref41] Walker JT, Bradshaw DJ, Bennett AM, Fulford MR, Martin MV, Marsh PD (2000). Microbial biofilm formation and contamination of dental-unit water systems in general dental practice. Appl Environ Microbiol.

[ref42] Weavers L (1999). Disinfetion and sterilization using ozone. Disinfect Steriliz Preserv.

[ref43] Wirthlin MR, Marshall GW (2001). Evaluation of ultrasonic scaling unit waterline contamination after use of chlorine dioxide mouthrinse lavage. J Periodontol.

[ref44] Zanetti F, De Luca G, Sacchetti R (2009). Control of bacterial contamination in microfiltered water dispensers (MWDs) by disinfection. Int J Food Microbiol.

